# Combined sodium Dimercaptopropanesulfonate and zinc versus D-penicillamine as first-line therapy for neurological Wilson’s disease

**DOI:** 10.1186/s12883-020-01827-9

**Published:** 2020-06-27

**Authors:** Jing Zhang, Lulu Xiao, Wenming Yang

**Affiliations:** 1 Department of Neurology, First Affiliated hospital of Anhui University of Traditional Chinese Medicine, Meishan Road 117, Hefei, 230031 China; 2 Department of Neurology, Jinling Hospital, Medical School of Nanjing University, 305 East Zhongshan Road, Nanjing, 210002 Jiangsu Province China

**Keywords:** Wilson’s disease (WD), Sodium 2, 3-dimercapto-1-propane sulfonate (DMPS), D-penicillamine (DPA), Zinc, Global assessment scale (GAS), Barthel index

## Abstract

**Background:**

Even though recent research has achieved significant advancement in the development of therapeutic approaches for Wilson’s diseases (WD), the current treatment options available for WD are still limited, especially for WD patients with neurological symptoms. This study is intended to compare the therapeutic approaches for WD patients with neurological symptoms receiving either combined sodium 2, 3-dimercapto-1-propane sulfonate (DMPS) and zinc treatment or D-penicillamine (DPA) monotherapy as first-line therapy, and identify the more effective therapeutic approach.

**Methods:**

The case records of 158 patients diagnosed with neurological WD were retrospectively analyzed. These patients treated with intravenous DMPS + Zinc and in combination with oral zinc as a maintenance therapy (Group 1) or DPA alone (Group 2) for 1 year. During the period of treatment, the neurological symptoms of the patients were assessed using the Global Assessment Scale (GAS) and Barthel index. The key hematological and biochemical parameters of the patients (such as the levels of aminotransferase, serum ceruloplasmin, 24-h urine copper excretion), as well as adverse effects were recorded and analyzed.

**Results:**

Ninety-three patients in Group 1, displayed decreased GAS scores and increased Barthel indexes consistently in comparison with the baseline (*P* < 0.01). Among them, 82 patients (88.2%) exhibited significant neurological improvement after 1 year, while 8 patients (8.6%) experienced neurological deterioration. Among the 65 patients in Group 2, 37 patients (58.5%) exhibited neurological improvements, while 17 patients (26.2%) experienced neurological deterioration after 1-year follow up. Six patients discontinued their treatment midway due to their exacerbating neurological symptoms. A comprehensive comparison of the effectiveness of the two courses of treatment revealed that patients in group 1 demonstrated a higher improvement ratio (*P* < 0.01) and lower worsening ratio of the neurological symptoms for the patients (*P* < 0.01) in comparison to the patients in group 2. Meanwhile, renal function, liver enzyme and blood cell counts remained stabilized in group1.

**Conclusions:**

This study indicates that the combined therapeutic approach of DPMS and zinc may be a preferred first-line therapy in treating the neurological symptoms of WD, in comparison to the treatment with DPA.

## Background

Wilson’s disease (WD) is a rare, genetic disorder in which the metabolism of copper is impaired. Mutations in a gene, called ATP7B, which encodes for a protein involved in copper-transportation, forms the genetic basis of WD. Thus, Copper accumulation in different parts of the body is the primary pathological manifestations of WD. The accumulation of copper primarily affects the liver, brain, and cornea. This leads to significant liver damage, as well as neurological and psychiatric symptoms. Neurological symptoms in WD are often accompanied by movement disorders or behavioral abnormalities [1]. If untreated or delayed, it would be fatal within a few years from the onset of symptoms [2, 3].

Even though recent research has achieved significant advancements in the development of therapeutic approaches for WD, the options of current pharmacological treatments available for WD are still limited, especially for WD patients with neurological symptoms [4]. Copper chelators like D-penicillamine (DPA), trientine, sodium 2, 3-dimercapto-1-propane sulfonate (DMPS) and zinc salts are the current initial therapies available for WD. DPA, a copper chelator, is the first oral treatment offered after the diagnosis of WD, and trientine is another copper chelator, which is a commonly used alternative to DPA for patients intolerant to DPA [5]. Although the clinical benefits of treatment with DPA have been documented in detail, the serious side-effects and the initial exacerbation of the neurological symptoms, reported in 10–50% of patients with neurological WD, have vastly limited its widespread application [6, 7]. Trientine also has similar side-effects on the patients. In addition, its use is limited in developing countries due to its high cost and limited availability [8]. DMPS is a water-soluble chelating agent, which binds to the copper ions in order to form a thiol compound. This enables the DMPS to excrete the extra copper through urine after filtration in the kidneys [9]. Although it is not widely popularized in other areas, DMPS has been universally used as a routine treatment for WD in China for several decades [10, 11]. In addition, it is also commonly used as an alternative treatment for WD patients, who are intolerant to the DPA treatment [9, 12]. Zinc has been considered to exhibit a relatively moderate anti-copper effect, which makes it an effective and safe therapeutic agent for maintenance after the initial chelator therapy or as a first-line therapeutic for asymptomatic, presymptomatic, and even neurological patients [13–15].

Since WD is a rare genetic disease, it has been challenging to develop the best and most effective therapeutic approach for treating WD. In addition, the low incidence and heterogeneous clinical presentation of the disease make it very difficult to design a randomized controlled trial. Thus, the degree of efficiency and safety of existing treatment options have been developed from studies based on clinical patients, which makes it complicated to devise definitive recommendations [16].

Until now, the investigations of the combined therapeutic approach of DMPS + zinc and DPA monotherapy in WD patients who manifested neurological symptoms are limited. Therefore, the aim of this study was to fill the scientific gaps in this field of research by comparing the two different therapeutic approaches and identifying the more effective therapeutic approach.

## Methods

### Patient information

In this retrospective study, the data of 158 WD patients who demonstrated neurological symptoms, was collected from the Department of Neurology at the first affiliated hospital of Anhui University of Traditional Chinese Medicine, during the time period of July 2015 to June 2018. Only those patients who had been recently diagnosed and had not received any initial treatment, were selected to be included in this study.

The diagnosis of the disease was based on the characteristic clinical manifestations of WD. It followed the following criteria: (1) positive family history; (2) the presence of K-F ring; (3) low serum ceruloplasmin levels (< 200 mg/l); (4) elevated 24-h urinary copper excretion (> 100 μg/24 h, and > 40 μg/24 h is suggestive in asymptomatic children); (5) elevated urinary copper excretion following the administration of the penicillamine (> 1600 μg/24 h). Evaluation of K-F ring on slit lamp was performed by experienced ophthalmologists.

All the medical details of the patients, including the demographic data, family history, medical history, and clinical features, were recorded in detail. The hematological and related biochemical criteria index [such as white blood cell (WBC) and platelet (PLT) counts, liver function and renal function, 24-h urine copper and ceruloplasmin (CER)] were all analyzed and recorded. Any adverse effects of both the treatments being compared were also recorded. In addition, the neurological symptoms of the patients were assessed by applying the neurological subscale of Global Assessment Scale (GAS) [17] and Barthel index [18].

### Treatment

#### Intravenous DMPS + zinc (group 1)

The initial dose at which DMPS was administrated to WD inpatients (*n* = 93) was 5-10 mg/kg per day. This dosage was then gradually increased to 20 mg/kg per day during a period of 1–2 weeks. This dosage was further maintained for 8 weeks (8 courses). Each treatment course comprised of 5 consecutive days of treatment with DMPS, and 2 days withdrawal period to promote tolerance [10]. After intravenous treatment was completed, zinc was given as the maintenance therapy for 1 year. In cases of zinc agents, the zinc dosage was administered as age-dependent. For patients < 15 years old, 75 mg/d of zinc element was divided into three doses over a period of 24 h. For older children and adults, 150 mg/d was administered with three doses over a period of 24 h.

#### DPA monotherapy (group 2)

The WD patients (*n* = 65) who were either intolerant to DMPS (e.g. allergic to DMPS) or refused hospitalization for the long term DMPS + Zinc treatment were assigned to group 2 and were treated with the daily DPA monotherapy. In case of DPA, an initial dose of 5 mg/kg/day was administered, following that it was gradually increased to 20 mg/kg/day during a period of 1–3 weeks.

In addition, all the patients were recommended to follow a special diet with low copper. The follow-up checkups of the patients were conducted at 2 weeks, 4 weeks, 8 weeks and 1 year. During the period of the treatment, 6 patients quitting the DPA treatment and adjusting to DMPS therapy withdrew from the study midway due to the exacerbating neurological symptoms.

### Statistical analysis

All statistical analysis was performed by using the SPSS software version 22.0 (International Business Machines Corporation, Armonk, NY, USA). The results have been demonstrated as mean ± SD or as the median and range. The significance of the data obtained in this study was analyzed through *t* tests for quantitative data and χ2 tests for qualitative data. All *P* values were based on two-tailed comparisons and those less than 0.05 were considered statistically significant.

## Results

### Baseline patient characteristics

The primary characteristics of the patients prior to initiation of treatment are shown in Table 1. Ninety-three patients (with mean age of 24.47 ± 8.00 years), out of which 64.5% were male, were assigned to group 1. These patients were scheduled to receive intravenous DMPS in combination with zinc as the first-line therapy. Sixty-five patients (with mean age of 26.93 ± 8.16 years), out of which 50.8% were male, were assigned to group 2. They were scheduled to receive DPA monotherapy as the first-line therapy. All of the patients included in this study demonstrated manifestations of neurological symptoms, which included dystonia (100 cases, 63.3%), dysarthria (75 cases, 47.5%), salivation (81 cases, 51.2%), dysphagia (45 cases, 28.5%), tremor (108 cases, 68.4%), parkinsonism (89 cases, 56.3%), ataxia (45 cases, 28.5%), and epileptic seizures (5 cases, 3.2%). After the first 4-week treatment, 6 patients quitted the DPA treatment and adjusted to DMPS therapy for the emerging severe neurological symptoms.

**Table 1:** Characteristics of the study population stratified by chelation therapy

	Group 1 (*N* = 93)	Group 2 (*N* = 65)	*P* value
Age (years)	24.47 ± 8.00	26.93 ± 8.16	0.062
Female (*n*, %)	33 (35.5%)	32 (49.2%)	0.084
Time since first symptoms (months)	24 (3–60)	23 (3–96)	0.748
Psychiatric symptoms (*n*, %)	5 (5.4%)	4 (6.1%)	0.836
Hepatic dysfunction at diagnosis (*n*, %)	23 (24.7%)	19 (29.2%)	0.529
Kayser-Fleischer corneal rings (n, %)	93 (100%)	65 (100%)	
Serum ceruloplasmin (< 200 mg/L)	93 (100%)	65 (100%)	
Abnormal brain MRI (*n*, %)	93 (100%)	65 (100%)	

Group 1: treated by sodium 2, 3-dimercapto-1-propane sulfonate in combination of zinc; Group 2: treated by D-penicillamine; MRI: magnetic resonance imaging

The baseline characteristics of patients in both the groups were relatively similar **(**Table 1**)**. In case of group 1, the average time since first symptoms was 24 months (ranging between 3 and 60 months), whereas in case of group 2, the average was 23 months (ranging between 3 and 96 months). All patients included in this study presented with the common hallmark syndromes of WD, like corneal K-F rings, low serum ceruloplasmin levels, high 24-h urine copper levels and abnormal MRI findings. Structural brain MRI of these patients revealed that the patients presented with widespread lesions throughout the brain. The scans produced high-signal intensity on T2 weighted images and low-intensity on T1 scan in the basal ganglia (144 case, 91.1%), thalamus (37 cases, 23.4%), midbrain (19 cases, 12%), pons (21 cases, 13.3%) and cerebellum (23 cases, 14.6%).

### Serum ceruloplasmin and 24-h urinary copper

The levels of serum ceruloplasmin (normal range is 200-600 mg/l) and 24-h urine copper (normal ranges < 100 μg/24 h) of all patients during the period of this study are presented in Table 2. In case of both groups, the levels of serum ceruloplasmin were not significantly different in comparison to the baseline levels (*P* > 0.05). Comparison between the two groups showed that there was no statistically significant difference, in the serum ceruloplasmin levels between the two therapeutic approaches at the 1-year follow up (*P* > 0.05). After 2 weeks of treatment, it was observed that the 24-h urinary copper excretion sharply increased in patients of either group (*P* < 0.01), and then gradually decreased after 4 weeks of treatment; statistically significant differences were observed between the two groups at the 2-week follow up (*P* < 0.05).

**Table 2:** Serum ceruloplasmin and 24 h urine copper in all patients with Wilson’s disease during treatment

Indicator	Time	Group 1 (*N* = 93)	Group 2 (*N* = 65/59)	*P* value
Serum ceruloplasmin (mg/L)	Baseline	79.34 ± 18.10	84.62 ± 15.75	0.648
	8 weeks	82.24 ± 8.24	82.15 ± 15.89	0.965
	1 year	81.75 ± 7.63	84.17 ± 9.18	0.147
24-h urinary copper (ug/24 h)	Baseline	393.23 ± 213.94	395.16 ± 225.27	0.956
	2 weeks	1775.29 ± 665.92	1550.76 ± 540.06	0.026^*^
	4 weeks	1509.21 ± 567.69	1598.01 ± 797.11	0.415
	8 weeks	799.36 ± 279.42	874.01 ± 348.16	0.147
	1 year	168.77 ± 80.72	176.69 ± 90.64	0.585

Group 1: treated by sodium 2, 3-dimercapto-1-propane sulfonate in combination of zinc; Group 2: treated by D-penicillamine; Compared with Group 2, ^*^P < 0.05; In Group 2: Baseline, 2 and 4 weeks: *N* = 65, 8 weeks and 1 year: *N* = 59

### Neurological outcome

The temporal trends of neurological outcome of the WD patients are shown in Table 3. No significant differences were found between the GAS scores and Barthel indexes of the two groups at the baseline (*P* > 0.05). In comparison with the baseline, the GAS scores of the group 1 patients remained stable after 2 weeks of DMPS treatment (*P* > 0.05) and then gradually decreased after 8 weeks of treatment (*P* < 0.01). Correspondingly, the scores of Barthel indexes remained stable at 4 weeks of DMPS treatment (*P* > 0.05) and then gradually increased after 8 weeks of treatment (*P* < 0.01). The neurological outcome of the patients in group 1 was similar to the baseline after 2 weeks of treatment (*P* > 0.05). Thirty-seven patients (39.8%) exhibited improvements in their neurological symptoms after 4 weeks of treatment. By 8 weeks, the number of patients exhibiting neurological improvements increased to 64 (68.8%). By the 1-year follow up, the number increased to 82 patients (88.2%). However, 11 patients (11.8%) from group 1 presented deterioration in their neurological conditions after 4 weeks of treatment. In addition, most of these patients with neurological deterioration (8 out of 11) did not recover to the baseline by the 1-year follow up.

**Table 3:** Neurologic outcomes using the GAS scoring system and Barthel scores in all patients with Wilson’s disease during treatment

		Baseline	2 weeks	4 weeks	8 weeks	1 year
Group 1		*N* = 93	*N* = 93	*N* = 93	*N* = 93	*N* = 93
	**GAS scores**	8.74 ± 2.99	8.79 ± 2.90	8.46 ± 3.07	6.79 ± 2.27^**^	5.89 ± 2.11^**^
	Deterioration (n, %)		7/93 (7.52%)	11/93 (11.8%)	10/93 (10.8%)	8/93 (8.6%)
	Improvement (n, %)		9/93 (9.6%)	37/93 (39.8%)	64/93 (68.8%)	82/93 (88.2%)
	**Barthel scores**	54.52 ± 14.20	55.01 ± 14.71	56.99 ± 15.34	66.51 ± 15.51^**^	74.68 ± 16.15^**^
Group 2		*N* = 65	*N* = 65	*N* = 65	*N* = 59	*N* = 59
	**GAS scores**	8.40 ± 3.49	8.69 ± 3.44	9.20 ± 3.89^*^	8.17 ± 3.57	7.89 ± 3.76
	Deterioration (n, %)		13/65 (20%)	22/65 (33.8%)	21/65 (32.3%)	17/65 (26.2%)
	Improvement (n, %)		8/65 (12.30%)	19/65 (29.2%)	30/65 (46.1%)	37/65 (58.5%)
	**Barthel scores**	58.38 ± 18.65	57.08 ± 17.92	53.23 ± 19.27	61.52 ± 18.67	64.57 ± 21.27

Group 1: treated by sodium 2, 3-dimercapto-1-propane sulfonate in combination of zinc; Group 2: treated by D-penicillamine; Global Assessment Scale, *GAS* Compared with baseline, ^*^P < 0.05, ^**^P < 0.01

No patient demonstrated neurological deterioration and 18 patients (18/93) gradually exhibited neurological improvements during zinc maintenance therapy, as indicated by an increase of 1–2 points on the GAS.

In case of patients in group 2, the GAS scores were significantly higher compared to the baseline in the first 4 weeks after treatment (*P* < 0.05). By 8 weeks pot treatment, the GAS scores of the patients were slightly decreased (*P* > 0.05). Compared with the baseline, the Barthel index scores in group 2 patients slightly decreased after 4 weeks of DPA monotherapy and gradually increased after 8 weeks of treatment, and no differences were found during all the follow-up times (*P* > 0.05). In this group, after the first 4-weeks of DPA monotherapy, 22 patients (33.8%) exhibited significant neurological deterioration. Six of these patients quitted the DPA treatment regime due to the severe exacerbation of their neurological symptoms. By the 1-year follow up, 17 patients (26.2%) still exhibited significant neurological deterioration, whereas 37 patients (58.5%) demonstrated significant improvements in their neurological symptoms at the 1-year follow up post the DPA treatment.

Thus, the comparative analysis of the two groups demonstrated that after 1 year of therapy, the neurological improvement ratio of the patients in group 1 was significantly better than that of in group 2 (*P* < 0.01). In addition, the deterioration ratio in group 1 was remarkably lower than that of in group 2 (*P* < 0.01). It is noteworthy that some of the neurological symptoms like dysarthria, dysphagia and dystonia have a higher predisposition to deteriorate during the courses of both the therapeutic approaches compared in this study.

### Liver function and renal function

No significant differences were observed between the serum concentrations of alanine aminotransferase (ALT) and aspartate transaminase (AST) in two groups at baseline (34.85 ± 20.45 IU/L vs 36.45 ± 26.75 IU/L, *P* > 0.05; 28.43 ± 9.95 IU/L vs 31.37 ± 14.29 IU/L, *P* > 0.05)**.** After 4 weeks of combined therapy, the ALT and AST levels were mildly elevated in both groups in comparison to the baseline (*P* > 0.05). By 8 weeks post treatment, the ALT and AST levels decreased gradually in both groups (*P* > 0.05). No significant differences in the serum ALT or AST levels were observed between the WD patients of both groups by the 1-year follow up (30.37 ± 17.56 IU/L vs 29.66 ± 14.82 IU/L, *P* > 0.05; 26.95 ± 9.87 IU/L vs 28.42 ± 6.90 IU/L, *P* > 0.05).

There were also no significant differences in the serum levels of creatinine (Cr) and blood urea nitrogen (BUN) between the two groups at baseline (66.21 ± 16.92 IU/L vs 62.99 ± 15.65 μmol/L, *P* > 0.05; 5.49 ± 1.67 IU/L vs 5.05 ± 1.53 μmol/L, *P* > 0.05). The same was true at the 4-week, 8-week, and 1-year follow up (*P* > 0.05)*.* Renal function remained stable for patients in both groups throughout the study.

### Blood cell counts

No significant differences were discovered in the platelet (PLT) and white blood cell (WBC) counts of patients from both groups at baseline (*P* > 0.05). In case of both groups, the PLT counts remained stable throughout the period of the study, and no significant differences were observed between two groups (*P* > 0.05). The WBC counts in case of patients from group 1 were mildly decreased in comparison to the baseline by 2 and 4 weeks (5.01 ± 1.61*vs* 4.91 ± 1.29, *P* > 0.05; 5.01 ± 1.61 vs 4.85 ± 1.09, *P* < 0.05). By the 8-week follow up, the WBC counts gradually elevated and reached up to the baseline (5.01 ± 1.61*vs* 4.94 ± 1.13, *P* > 0.05). In case of group 2, the WBC counts of patients decreased in the first 4 weeks after treatment (4.97 ± 1.50 vs 4.47 ± 1.47, *P* < 0.01; 4.97 ± 1.50 vs 4.40 ± 1.61 *P* < 0.01); however, by the 1-year follow-up, it still failed to recover to baseline (4.97 ± 1.50 vs 4.45 ± 1.10, *P* < 0.01). Analysis of the differences in the WBC counts between the two groups at each follow up time point revealed that there were significant differences between the two groups at the 2-week, 4-week, 8-week, and 1-year follow up time points (4.91 ± 1.29 vs 4.47 ± 1.47, *P* < 0.05; 4.85 ± 1.09 vs 4.40 ± 1.61, *P* < 0.05; 4.94 ± 1.13 vs 4.63 ± 1.45, *P* < 0.05; 4.92 ± 1.09 vs 4.45 ± 1.10, *P* < 0.05).

### Neuroimaging data

**Fig. 1:**
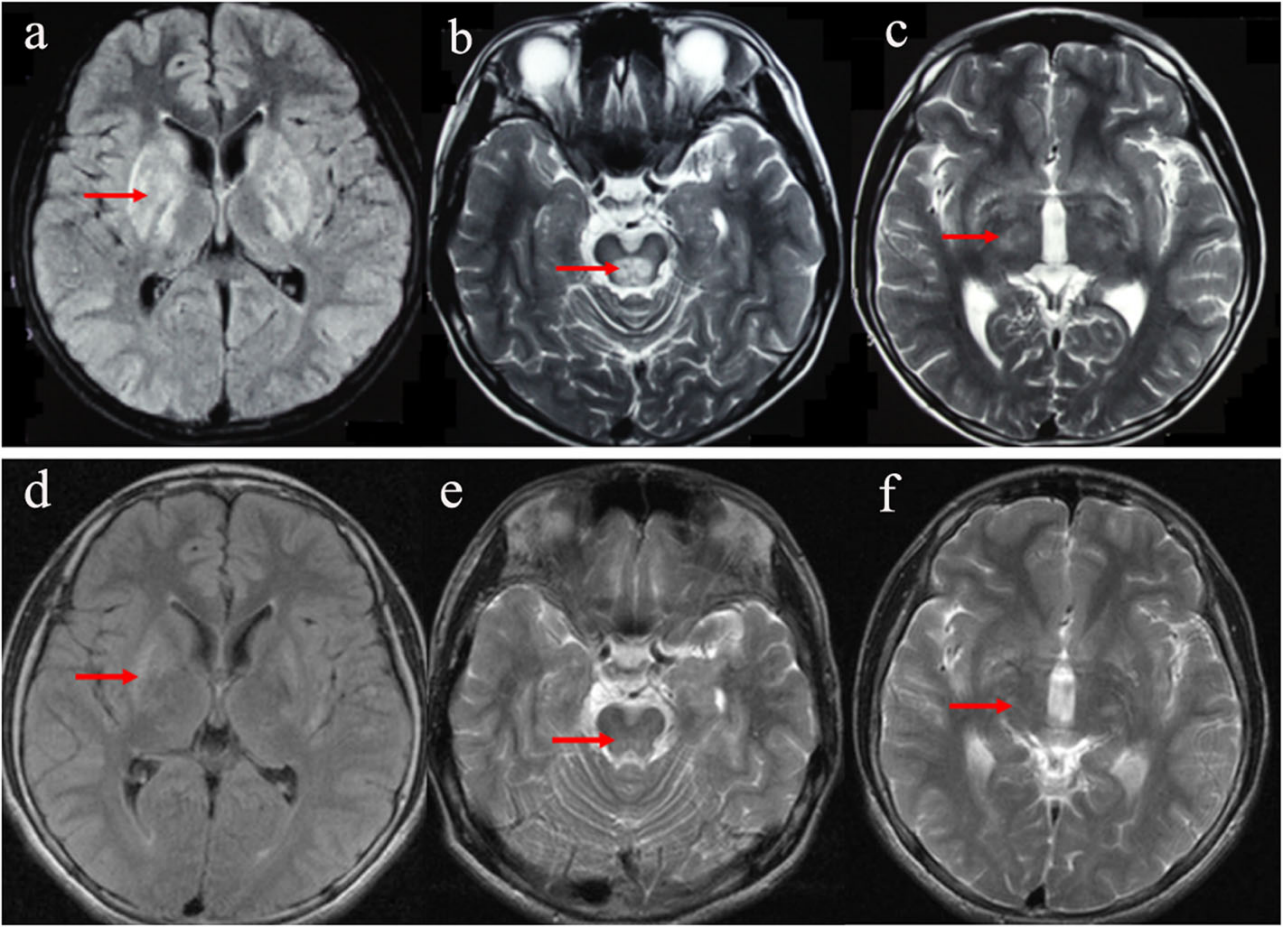
Brain magnetic resonance imaging (MRI) changes in a patient with Wilson’s disease prior to and after 1 year of treatment. Compared with prior treatment (**a** − **c**), abnormal signal intensities in basal ganglia, thalamus, and midbrain were all obviously altered after 1 year of combined therapy (**d** − **f**)

All patients with neurological manifestations of WD exhibited brain pathologies on baseline MRI. In group 1, 45 patients (45/93, 48.4%) exhibited significant improvements after 1 year of combined treatment, with abnormal signal intensities decreasing or disappearing (Fig. 1). In group 2, 20 patients (20/65, 30.8%) exhibited significant changes after 1 year of treatment. Thus, the comparative analysis of the two groups demonstrated that after 1 year of therapy, the MRI improvement ratio of the patients in group 1 was significantly better than of those in group 2 (*P* < 0.05).

### Adverse effects

The main adverse effects recorded in case of patients from both groups were certain gastrointestinal symptoms, like pain in the abdomen, nausea, and vomiting. These symptoms could easily be alleviated by dosage adjustment and modifying the time line of the administration of the medicine. In addition, no gastrointestinal hemorrhage was recorded in patients of either group. In group 1, 6 patients with splenomegaly suffered from myelosuppression during DMPS therapy, 13 patients from group 1 suffered from the gastrointestinal symptoms during the oral zinc treatment, but most of those were alleviated by dosage adjustment or by the modification of medication schedule. In group 2, 10 patients suffered from the gastrointestinal symptoms discussed earlier. Five patients developed myelosuppression and this was not successfully alleviated after 1 year of the DPA treatment.

## Discussion

It has been challenging to develop an universally accepted therapeutic approach to address WD, due to its heterogeneous clinical manifestation [1]. Currently, the best therapeutic approach for treating each specific presentation of WD depends on different factors like the opinion of the physician, the availability of the drug and the patient acceptance. This study showed the efficacy and safety of a combination therapeutic approach in which DMPS + Zinc as an initial treatment for the WD patients manifesting neurological symptoms.

Furthermore, 88.2% of the patients receiving intravenous DMPS + Zinc as the first-line of therapy showed gradual neurological improvement after 1 year of treatment, while only 58.5% of the patients undergoing the DPA monotherapy group exhibited neurological improvements. This data demonstrates that the combinatorial therapeutic approach using DMPS and zinc as the initial therapy was significantly more effective than DPA monotherapy in case of WD patients manifesting neurological symptoms.

It has been reported that neurological symptoms deteriorate with the chelating agents due to the re-distribution of copper ions [19]. This study showed that only 8.6% of the patients in the combined therapy group presented with neurological deterioration at the 1-year follow up, however, in the DPA monotherapy group, 26.2% of patients demonstrated neurological deterioration. This demonstrates that the combined therapy is safer than that of the DPA monotherapy as an initial treatment in patients with neurological WD. In case of most of the patients, hepatic function improved slightly and kept constant after treatment. Additionally, the WBC and PLT counts remained stable in the combined therapy group during the 1 year of follow-up time. This indicates that there was no hematological toxicity in the treatment regimes.

Studies have shown that DMPS is one of the best copper-chelators used for medicinal purposes. This can be attributed to its characteristic of a very low probability to cause allergic reactions, low hematological toxicity and its efficiency in excreting copper from the patient’s body [9]. This study showed that the 24-h urine copper levels increased significantly during intravenous DMPS treatment without having any adverse effect on the platelet counts and renal function of the patients. However, intravenous DMPS is primarily used as a first-line treatment after the diagnosis of WD in order to effectively alleviate severe symptoms in acute stages of the disease. Once the initial symptoms have been alleviated, the patients are shifted to an oral medication therapy accompanied with a low copper diet.

In developing countries, DPA has been primarily used as a first-line drug for WD patients for several decades, for its powerful de-coppering capability. However, DPA is also prone to causing several adverse effects, including serious conditions like bone marrow depression and anemia/leucopenia. Studies have also shown that it can cause a potential deterioration of the neurological symptoms of WD [2, 20]. Zinc exhibits extremely low neurotoxicity and is considered safe in the treatment of WD patients. It has been recommended as first-line treatment for asymptomatic and pregnant patients and even neurological WD patients [15, 21, 22]. However, others still remain skeptical and consider that zinc alone is not a preferred method in treating WD with symptomatic patients, due to a relatively slow action to achieve a negative Cu balance, and should be recommended as maintenance treatment after decoppering with chelators [12, 23–25].Trientine is an efficient alternative when WD patients become intolerant to DPA, however, its high cost and limited availability has made its use in the developing countries vastly difficult [2]. Scientific reviews have reported that 24.4% of the patients treated with DPA based therapy display adverse effects, with the manifestation of severe side effects in about 50% of those patients [22].

This study showed that 33.8% of the patients treated with DPA monotherapy experienced exacerbated neurological symptoms by the 4-week follow up time point and no significant recovery (e.g., 17 out of 22) was recorded in most of the patients by the 1-year follow up time point. Besides, 6 excluded patients discontinued DPA treatment in the midway for severe adverse events and changed their therapeutic regime. Thus, the results of this study along with the published literature about DPA as a first-line treatment for WD suggests that DPA has a considerable amount of neurotoxic effect and some serious side effects, which make it a suboptimal first-line medical therapy for treating WD with neurological symptoms.

Numerous studies on several different groups of WD patients have demonstrated that zinc therapy is considerably safe and inexpensive [10, 22]. The mechanism of zinc chelates with copper is different from that of iron chelators. The mechanism of chelation by zinc involves inducing liver cell metallothionein and intestinal, which binds to copper with a great affinity and inhibits the transfer of copper into the portal circulation [5]. Since zinc has extremely low toxicity and is also cost effective, it is an optimal therapeutic choice. It has been successfully used as first-line treatment for treating pre-symptomatic and pregnant patients for maintaining their copper levels consistently, and in pediatric WD patients with hepatic presentation [26, 27].

Zinc monotherapy has been reported to be safe and efficient for neurological WD and is even recommended as first-line treatment for neurological WD patients [15, 21, 22]. In the Netherlands,10 patients with neurological symptoms were treated with zinc monotherapy, and 9 of them achieved good outcomes after 14-years follow up [15]. However, owing to its slow action in achieving a negative Cu balance, zinc therapy has been considered inefficient for rapid reversing neurological deficits and thereby preventing WD progression [6, 25]. It has been reported that 29.4% (5/17) of the neurological patients with a history of DPA intolerance that were treated with zinc monotherapy exhibit neurological deterioration, while only 11.8% (2/17) of them exhibit neurological improvements after 1 year. Noteworthily, the combination of DMPS and zinc as maintenance treatment was reported to be superior to that of zinc monotherapy in patients with neurological WD. Neurological deterioration was observed in 76.2% (17/21), while only 19.0% (4/21) of them exhibited neurological improvements after 1 year [12]. Meanwhile, the American Association for the Study of Liver Diseases (AASLD) recommend that the initial therapy of symptomatic WD patients should include a chelating agent [5]. In this study, 68.8% (64/93) neurological patients have achieved rapid improvement after only 8 weeks treatment, which indicates that DPMS as internal therapy can rapidly improve neurological symptoms. In addition, no patient demonstrated neurological deterioration and 18 patients gradually exhibited neurological improvements during zinc therapy, which indicates that zinc agent is safety and efficacy as maintenance therapy.

It has been reported that combined therapy using chelating agents and zinc for WD can cause the aggravation of adverse reactions. An analysis from a total of 17 combined therapy studies involving 1056 patients demonstrates that the combined therapy of penicillamine plus zinc sulfate can result in a significantly higher mortality rate [28].Yang et al. [29] have recommended that the dosing interval between zinc preparation and oral chelating agents should be more than 2 h. In our study, to avoid the aggravation of adverse reactions caused by combined therapy using chelating agents and zinc, zinc was given as supplementation in the DMPS withdrawal period and as maintenance therapy after eight courses of DMPS treatment. On the one hand, zinc supplementation after 5 consecutive days of treatment with DMPS can prevent zinc deficiency, which is caused by the application of chelating agents. On the other hand, the aggravation of adverse reactions due to combined therapy using chelating agents and zinc can be reduced and even avoided.

In the present study, this combined therapeutic approach significantly improved the neurological symptoms and quality of daily life of neurological WD patients, while lowering the incidences of neurological deterioration. The adverse effects of the combined therapeutic approach were relatively mild, which primarily included gastrointestinal symptoms and transient decrease in white blood cell counts. In case of most of the patients, hepatic function improved slightly and kept constant after treatment. The incidence of myelosuppression as a side effect of the DMPS and zinc combined therapeutic approach was quite low and all cases which presented with myelosuppression had splenomegaly previously. Gastrointestinal symptoms can be alleviated by dosage adjustment. The findings of this study suggest that the combined therapy is more efficient and relatively safer compared to the DPA monotherapy as the first-line therapeutic approach for treating neurological symptoms of WD.

Significant improvements on MRI were observed in 48.4% of patients receiving the combined therapeutic approach after 1-year treatment, while only 30.8% of the patients undergoing the DPA monotherapy group exhibited neurological improvements. Noteworthily, no obvious changes in MRI abnormal signal intensities were detected for the patients who demonstrated neurological deterioration in both groups after 1 year of therapy. Recently, susceptibility-weighted imaging (SWI) has been reported to be conducted effectively in the detection of the changes during the therapy of metal chelators in patients with Wilson disease [30].

In this study, none of the WD patients exhibited any psychiatric symptoms except 9 patients with mild psychiatric symptoms (anxiety and mild cognitive impairment). For these 9 WD patients, no specific treatments were given. During the treatment, most of these psychiatric symptoms gradually disappeared. For those with serious dystonia and poor life quality, consistent symptomatic treatment (e.g. anticholinergic drugs and baclofen) were given in both groups.

Dietary restriction is helpful in controlling copper excess [6]. Lifelong treatment, compliance with treatment, safety assessment, and concomitant medications are also very important for WD patients [31]. In addition, earlier diagnosis and therapy is of great significance for the prevention of neurological deterioration in WD disease. Delayed diagnosis is one of the most important factors that determines poor treatment outcome [5, 23, 32].

## Conclusions

The findings of this study indicate that the combined therapeutic approach of treatment with DPMS and zinc as a first-line therapy may be more optimal in comparison to the DPA monotherapy for the treatment of WD patients manifesting neurological symptoms. There are a few limitations to the scope of this study. The retrospective nature of data collection poses a key limitation to the introduction of diversity within the dataset. The devising of studies with multi-cycle therapies and longer follow-up periods would be useful to shed more light upon the clinical value and potential side effects of the combination therapy. Finally, the genetic aspects of both the therapeutic approaches compared in this study have not been analyzed.

## Data Availability

The datasets used and/or analyzed during the current study can be provided by the corresponding author on request.

## Abbreviations

WD: Wilson’s disease
DMPS: Sodium 2, 3-dimercapto-1-propane sulfonate
DPA: D-penicillamine
GAS: Global Assessment Scale
ALT: Alanine aminotransferase
AST: Aspartate transaminase
PLT: Platelet
WBC: White blood cell
